# Building a Natural Language Processing Artificial Intelligence to Predict Suicide-Related Events Based on Patient Portal Message Data

**DOI:** 10.1016/j.mcpdig.2023.09.001

**Published:** 2023-09-30

**Authors:** Archis R. Bhandarkar, Namrata Arya, Keldon K. Lin, Frederick North, Michelle J. Duvall, Nathaniel E. Miller, Jennifer L. Pecina

**Affiliations:** a Mayo Clinic Alix School of Medicine, Rochester, MN; b Mayo Clinic Alix School of Medicine, Scottsdale, AZ; c Division of Community Internal Medicine, Department of Medicine, Mayo Clinic, Rochester, MN; d Department of Family Medicine, Employee and Community Health, Mayo Clinic, Rochester, MN

## Abstract

**Objective:**

To develop a natural language processing artificial intelligence model trained on text from patient portal messages to predict 30-day suicide-related events (SRE).

**Patients and Methods:**

Patient portal messages sent by patients between January 1, 2013, and October 31, 2017 were screened for an associated SRE within 30 days. For both patient portal messages associated with a 30-day SRE and a randomized control set, we automatically extracted several features: (1) frequencies of keywords; (2) message metadata; and (3) message sentiment.

**Results:**

A total of 840 patient portal messages were included in our final analysis, including 420 messages with and without an associated 30-day SRE. Patient messages with an associated 30-day SRE had a mean sentiment score that was less than those without an SRE (*P*<.001). Messages with an associated 30-day SRE had greater word counts (*P*=.002) and more use of ellipses (*P*=.02), but less use of exclamation marks (*P*=.04) and question marks (*P*=.007) compared with messages without a 30-day SRE. The neural network machine learning model had the highest area under the receiver operating curve at 0.710, with a sensitivity of 56.0% and a specificity of 69.0%.

**Conclusion:**

A natural language processing artificial intelligence model trained on a subset of patient portal message data was able to predict 30-day SRE at a level comparable to commonly used suicide assessment tools. Predictors that conveyed the overall tone of a patient message, such as the sentiment score, were more highly weighted by machine learning models in predicting 30-day SRE than the frequencies of individual words.

Over the past few years, there has been a rapid growth in telemedicine utilization in primary care owing to the temporary shutdown of outpatient in-person practices during the COVID-19 pandemic.^1^, ^2^, ^3^ In one national survey of primary care physicians, telemedicine utilization grew from 5% before the pandemic to 46% during the pandemic.^2^ Electronic patient portals are one form of telemedicine service in which care can be delivered in an asynchronous, virtual format through a secure electronic chat between patients and providers. Although evidence is mixed with regards to the effect of electronic patient portals on health care outcomes,^4^ use of electronic patient portals has been increasing even before the COVID-19 pandemic with a previous study at our institution showing an average increase of 110% in the amount of primary care provider message responses from 2013 through 2018.^5^ In our practice at Mayo Clinic, one study found that 2 million patient portal messages were generated over a 7 month window during the COVID-19 pandemic.^6^ As electronic patient portals continue to grow in utilization, there is both an opportunity and a need to harness the massive data generated from patient portals to improve the delivery of primary care.

Alongside the expansion of electronic patient portals and the large amount of data generated from them, there has been a remarkable growth in artificial intelligence (AI) with the ability to process unstructured text. This ability, known as natural language processing (NLP), has been documented in stunning fashion in recently released generative large language models such as Chat generative pre-trained transformer (ChatGPT) and GPT-4.^7^ For example, ChatGPT has recently been shown to perform at a passing threshold on all stages of the US medical licensing exam.^8^ By contrast, predictive language AI models use various types of input to predict future events. The dramatic rise of these new forms of NLP AI has led to multiple calls that any deployment of the technology in health care be done so responsibly in a way that is safe for patients.^9^ Key safety concerns of modern general NLP AI include an inability to identify context-specific clues or discriminate between reliable and unreliable sources of information. These concerns found the need for NLP AI to be constructed in a way that is context-specific, with model training vetted by health care providers before being integrated with electronic patient portals.

One context that is ripe for deploying NLP AI to assist providers is with delivering mental health care through the electronic patient portal, specifically with regards to identifying patients at high risk of suicidal behavior. Several studies have reported predictive models on the basis of data from either social media or text messages which have considerable accuracy in predicting imminent suicidal risk.^10^^,^^11^ In the same way that analyses have shown “digital biomarkers” of changing utilization patterns in social media and text messages before suicidal behavior, it is possible that patients may show patterns of language use before a suicide-related event (SRE) in a way that can be detected by an NLP AI.

In this study, we sought to develop an NLP AI trained on deidentified unstructured text data from patient portal messages to predict 30-day SREs defined as having either an emergency department or inpatient encounter for unipolar depression, suicidal ideation (SI), suicide attempt, or death by suicide.

## Methods

### Data Collection

As described previously by our group,^12^ data collection from the Mayo Clinic patient portal dataset was performed in accordance with Mayo Clinic Institutional Review Board protocol 18-000664. All patient portal messages sent by patients between January 1, 2013, and October 31, 2017 were reviewed for any patients who had an SRE within 30 days after sending their portal message. An SRE was defined as having either an emergency department or inpatient encounter for unipolar depression, SI, suicide attempt, or death by suicide. Messages were included if they were sent by a patient with an address in one of the 8 counties served by our regional medical examiner’s office. Patient portal message dates and content were collected from the Mayo Clinic patient portal dataset. Deaths by suicide were determined from medical examiners’ data from the 8 regional counties included in our analysis. We identified patients admitted to the emergency department or hospital by using an electronic medical record search tool, called the Mayo Data Explorer, to search for hospitalizations and emergency department visits for International Classification of Diseases codes for major depression, depressive disorder, suicide attempt, SI, and suicide and self-inflicted injuries. For any patients with a 30-day SRE, we then reviewed the electronic medical record manually to confirm that the emergency department visit and/or hospitalization was for depression, SI, and/or suicide attempt.

After identification of all patient portal messages in our dataset with an associated 30-day SRE, we randomly selected an equal number of patient portal messages without an associated 30-day SRE. An equal number of control messages were selected to avoid class imbalance in the process of training our machine learning models, as described below.^13^ All patient portal message text was manually deidentified of any personal health information by deleting identifiers before passing through our NLP pipeline.

#### Natural Language Processing Pipeline

A standard “bag-of-words” approach was used in our NLP pipeline for analyzing patient portal message text, which was implemented in R version 4.2.1.^14^, ^15^, ^16^ Features extracted from patient portal message data included the frequencies of stemmed keywords (eg, depress); metadata related to punctuation and the length of the message; and the average sentiment of the message. For each message, the subject and content of the message were concatenated into a single string object. The R packages “tm" and “SnowballC” were used to automatically remove stop words and stem individual words in the patient portal text. Stop words are words that do not contribute to the semantic meaning of text, such as “the,” “is,” and “and.” Stemming is the process of linking together words that convey similar semantic meaning. For example, the words “depression,” “depressing,” and “depressed” would be stemmed in our pipeline to the same stemmed keyword of “depress.” A document-term matrix was then created to represent the frequencies of individual stemmed keywords in patient portal messages. A sparsity filter was applied to reduce the size of the document-term matrix, in which tokenized keywords with a sparsity of greater than 99% in the overall text were removed. The R package “stringr” was used to calculate metadata such as percentage of punctuation (periods, question marks, and exclamation marks) that were exclamation or question marks, presence of ellipsis (“...”), percentage of alphabet characters that were capital letters, and number of words in the deidentified portal message.

Sentiment analysis using the “sentimentr” R package was used to calculate the average sentiment per sentence of portal messages.^15^ The particular form of sentiment analysis implemented by this R package uses a weighted dictionary of polarized words that have been annotated to have positive and negative connotations. Individual words have a score bounded between a negative one (negative sentiment) and a positive one (positive sentiment), and a sentiment score on the basis of a weighted sum of polarized words is calculated for each sentence.

#### Machine Learning Modeling Process

Machine learning models were created to associate patient portal message metadata and the frequencies of stemmed keywords with whether an SRE event occurred.^16^ The R packages “caret” and “MLeval” were used to train different machine learning algorithms and evaluate their performance. The random forest, neural network, bagged decision tree, and extreme gradient boosting algorithms were selected to represent 4 different machine learning algorithms with different model architectures and convergence properties. Model input included the frequencies of 550 stemmed keywords and 6 other features (mean message sentiment, number of words, proportion of capitalized letters, proportion of exclamation marks, proportion of question marks, and presence of an ellipsis). All model features were normalized to have a mean of 0 and a variance of 1 so that the different models would compare each feature on the same scale. Of the 820 total patient portal messages included in the sample, 672 (80%) messages were used for training our machine learning models, and 168 (20%) messages were used to compute model performance.. Models were trained using cross-validation in a 10-fold, 5-repeat fashion. Metrics of model performance included sensitivity, specificity, positive predictive value, negative predictive value, and area under the receiver operating curve (AUC-ROC). Explainability analysis was performed using the variable importance function in the “caret” package, which is capable of computing model-specific importance metrics for individual machine learning algorithms. Variable importance metrics are out of 100, with features having an importance value closer to 100 being weighted more heavily in the model’s final prediction.

## Results

### Message Characteristics

A total of 840 patient portal messages were included in our final analysis, including 420 messages with an associated 30-day SRE and 420 messages without an associated 30-day SRE. Our cohort of SRE messages included 7 messages from 3 patients who died by suicide, 56 messages from 23 patients who attempted suicide, and 357 messages from 126 patients who were hospitalized or seen in the emergency department for depression and/or SI without a suicide attempt. Patient messages with an associated 30-day SRE had a mean sentiment score (+0.02±0.16) that was on average less compared with those without an SRE (+0.06±0.16) (*P*<.001). The number of words in messages from patients with an associated 30-day SRE was on average greater at 81.2 words (SD=72.0 words) compared with an average of 66.5 words (SD=63.2 words) (*P*=.002) among the messages without an associated 30-day SRE. The proportion of exclamation marks (0.035 vs 0.055; *P*=.034) and question marks (0.115 vs 0.156; *P*=.007) was less in the group with an associated with 30-day SRE compared with those without. There was a greater presence of ellipses (“…”) in the messages with SRE (n=27, 6.4%) vs those without an associated SRE (n=13, 3.1%) (*P*= .02). There was no statistically significant difference in the proportion of capital letters (*P*= 66) (Table 1).

**Table 1:** Comparison of Patient Portal Message Characteristics With and Without a 30-Day SRE.

Characteristic	Message Without SRE (n=420)	Message With SRE (n=420)	Total (N=840)	*P*
Average sentiment score per sentence				<.001
Mean ± SD	+0.06±0.16	+0.02±0.16	+0.04±0.16	
Range	−0.65 to +0.78	−0.55 to +0.53	−0.65 to +0.78	
Number of words				.002
Mean ± SD	66.5±63.2	81.2±72.0	73.9±68.1	
Range	2-451	2-468	2-468	
Proportion of capital letters				.66
Mean ± SD	0.047±0.075	0.045±0.070	0.046±0.073	
Proportion of exclamation marks in punctuation				.04
Mean ± SD	0.055±0.146	0.035±0.117	0.045±0.132	
Not Applicable	9	19	28	
Proportion of question marks in punctuation				.007
Mean ± SD	0.156±0.237	0.115±0.202	0.136±0.221	
Not Applicable	9	19	28	
Presence of ellipsis (“...”)				.02
N (%)	13 (3.1%)	27 (6.4%)	40 (4.8%)	
Frequency of “depress” (mentions / message)				.004
Mean ± SD	0.045±0.302	0.114±0.386	0.080±0.348	
Frequency of “thank” (mentions or message)				.08
Mean ± SD	0.055±0.248	0.088±0.300	0.071±0.276	
Frequency of “help” (mentions or message)				<.001
Mean ± SD	0.164±0.436	0.302±0.649	0.233±0.557	
Frequency of “suicid” (mentions or message)				<.001
Mean ± SD	0.000±0.000	0.038±0.204	0.019±0.145	
Frequency of “anxieti” (mentions or message)				.32
Mean ± SD	0.000±0.000	0.002±0.049	0.001±0.035	
Frequency of “reall” (mentions or message)				<.001
Mean ± SD	0.074±0.296	0.174±0.449	0.124±0.383	

SRE, suicide-related event.

There were several stemmed keywords that were mentioned more frequently in messages with an SRE vs those without. The stemmed keyword “depress” had a frequency of 0.114 mentions per message (SD=0.386) among messages with an SRE compared with 0.045 mentions per message (SD=0.302) among messages without an SRE (*P*=.004). The stemmed keyword “help” appeared more frequently in messages with an SRE at 0.302 mentions per message (SD=.649) compared with 0.164 mentions per message (SD=.436) in messages without an SRE (*P*<.001). The keyword “suicid” was mentioned in 15 out of 420 messages with an associated 30-day SRE and in none of the messages without an SRE (Table 1).

#### Machine Learning Model Performance

**Figure 1:**
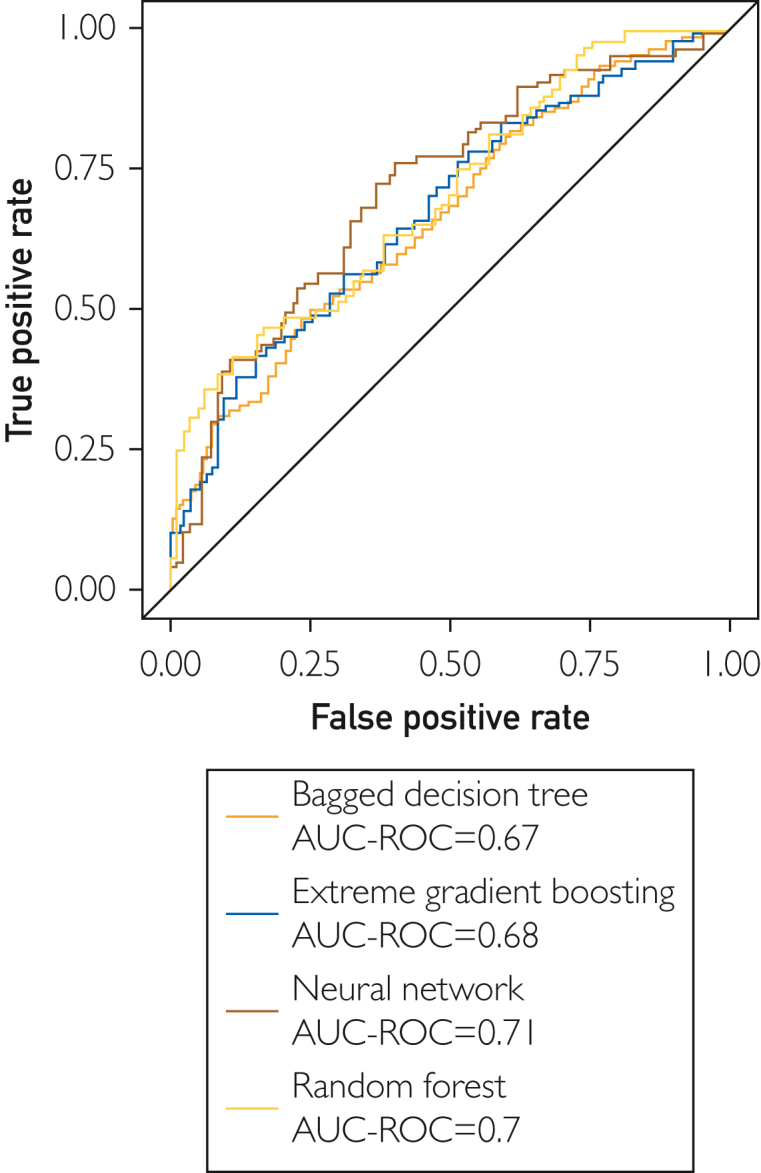
Comparison of receiver operating curves plotting true positive vs false positive rates among different machine learning algorithms on validation data (random forest, neural network, and bagged decision tree).

The training cohort for our machine learning models comprised an 80% sample of our total of 840 patient portal messages. Of the 3 machine learning models implemented, the neural network model had the highest AUC-ROC at 0.710 (95% confidence interval [CI], 0.63-0.79), followed by the random forest model at 0.700 (95% CI, 0.62-0.78), the extreme gradient boosting model at 0.680 (95% CI, 0.60-0.76), and the bagged decision tree model at 0.670 (95% CI, 0.59-0.75). (Table 2; Figure 1). The model with the highest sensitivity was the extreme gradient boosting model at 61.9% (95% CI, 0.51-0.72), followed by the bagged decision model at 57.1% (95% CI, 0.46-0.67), the random forest model at 56.0% (95% CI, 0.45-0.66), and the neural network model at 56.0% (95% CI, 0.45-0.66). The model with the highest specificity was the neural network model at 69.0% (95% CI, 0.59-0.78), then the random forest model at 65.5% (95%CI: 0.55-0.75), the bagged decision tree model at 61.9% (95% CI, 0.51-0.72), and the extreme gradient boosting model at 60.7% (95% CI, 0.50-0.70) (Table 2).

**Table 2:** Performance of Different Machine Learning Models in Predicting 30-Day SREs From Patient Portal Message Features (95% CI reported in parentheses)

Models	SEN	SPEC	PPV	NPV	AUC-ROC
Random forest	0.560 (0.45-0.66)	0.655 (0.55-0.75)	0.618 (0.51-0.72)	0.598 (0.50-0.69)	0.700 (0.62-0.78)
Neural network	0.560 (0.45-0.66)	0.690 (0.59-0.78)	0.644 (0.53-0.74)	0.611 (0.51-0.70)	0.710 (0.63-0.79)
Bagged decision tree	0.571 (0.46-0.67)	0.619 (0.51-0.72)	0.600 (0.49-0.70)	0.591 (0.49-0.69)	0.670 (0.59-0.75)
Extreme gradient boosting	0.619 (0.51-0.72)	0.607 (0.50-0.70)	0.612 (0.51-0.71)	0.614 (0.51-0.71)	0.680 (0.60-0.76)

AUC-ROC, area under the receiver operating curve; NPV, negative predictive value; PPV, positive predictive value; SEN, sensitivity; SPEC, specificity.

**Figure 2:**
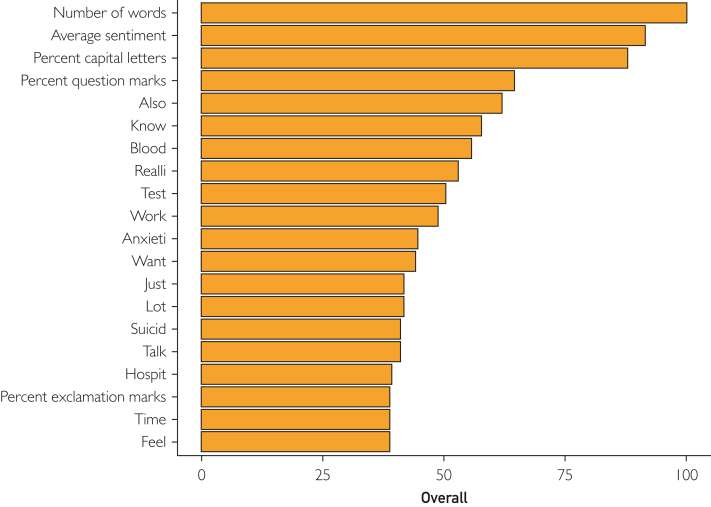
Explainability analysis reporting top features in random forest machine learning model.

Our AI explainability analysis found which features were most important in each machine learning model and which features had similar importance in all models. The average sentiment score was among the top 3 features in all machine learning models, with a variable importance score (VIS) of 91.5 (second most important feature) in the random forest model, 94.6 (third most important feature) in the neural network model, 100.0 in the bagged decision tree model, and 100.0 in the extreme gradient boosting model. The top 3 features in the random forest model were the number of words (VIS=100.0), average sentiment score (VIS=91.5), and percent capital letters (VIS=88.0). The top 3 features in the neural network model were the keywords “know” (VIS=100.0), “also” (VIS=98.6), and the sentiment score (VIS=94.6). The top 3 features in bagged decision tree model were the average sentiment score (VIS=100.0), proportion of capital letters (VIS=93.3), and number of words (VIS=89.5). The top 3 features in the extreme gradient boosting model were the sentiment score (VIS=100.0), proportion of capital letters (VIS=93.3), and number of words (VIS=65.1) (Figure 2).

## Discussion

The early detection and prevention of suicide and SREs remains a critical area of public health research. In this study, we developed an NLP AI capable of predicting 30-day SREs from features in the unstructured text data found in patient portal messages. Our highest-performing model defined by the greatest AUC-ROC was the neural network model, which had the highest AUC-ROC at 0.710 (95% CI, 0.63-0.79), a sensitivity of 56.0% (95% CI, 0.45-0.66), and specificity of 69.0% (95% CI, 0.59-0.78). Of note, these performance metrics were obtained with the limited amount of data used to train our model in this proof-of-concept study. With more data, model performance will likely improve, and it is possible that the highest-performing model may change. However, the model in its current state does perform roughly at or above the benchmark of commonly used suicide assessment tools such as the SAD-PERSONS scale. Although the reported performance of the SAD-PERSONS scale varies from study to study, 1 study in a large cohort of over 5000 adults reported an area under the curve (AUC) of 0.59 (95% CI, 0.51-0.67) of the modified SAD-PERSONS scale in predicting suicide death at one year.^17^ In addition, prospective analysis of the Columbia-Suicide Severity Rating Scale indicates that this instrument has limited predictive ability for future suicidal events (AUC 0.65; 95% CI, 0.60-0.69).^18^ These findings highlight the urgent need for improved predictive models for SREs.

Previous research on implementing NLP AI for the purpose of early detection of SREs has focused on mining social media and electronic health record data.^19^ With more than 80% of adults reporting active usage of social media, the massive amounts of unstructured text data generated by the usage of these platforms demonstrate significant potential for the prediction of SREs.^11^ Recent studies have shown promising results in leveraging social media data to predict and identify individuals at risk of SI. For example, a recent machine learning analysis found that a random forest model trained on regional Twitter data collected over 2 years predicted tweets expressing SI with an AUC of 0.88 (95% CI, 0.86-0.90).^20^ Similar models trained on Instagram usage in adolescents predicted short-term SIs with an AUC of 0.775.^21^ These studies report the feasibility of determining suicide risk by analyzing patterns in everyday social media usage that might not be readily detected in a routine health care appointment. Given the success of these predictive models, the results of our proof-of-concept study suggest that a similar approach can also be applied to patient portal messages.

Compared with previous studies that trained NLP AI models on social media data or clinical notes, our analysis is unique in that its dataset is on the basis of patient-generated messages on a secure electronic portal. There are several key insights to be gained from our univariate analysis comparing features in the patient portal messages that had 30-day SREs vs those that did not. Firstly, the average sentiment per sentence score stands out as a feature that was significantly different between the messages that had an SRE and those that did not. Notably, our analysis found that messages that had a 30-day SRE had significantly lower sentiment scores compared with those that did not. In a similar study, George et al^21^ found that sentiment analysis alone performed on psychiatry clinical notes could differentiate between nonsuicidal and suicidal cases. Of note, their high performing sentiment analysis-based predictive model had an AUC of 0.80 and was on the basis of a custom lexicon. In comparison, the sentiment scores calculated in our study were on the basis of a general library of polarized words in the “sentimentr” package, and our model performance could improve if we were to design a custom lexicon as well. Together, these findings suggest the potential utility of sentiment scores in clinical predictive models.

A unique aspect of the “bag-of-words” NLP model chosen in this study is that it lends itself to explainability analysis easier than most large language models. Our explainability analyses of the different machine learning models chosen for this study revealed several key insights into our model. First, it is apparent that the different models converged on different features to weight highest in their predictions. Despite the differences in the subset of top features, the sentiment score was among the top 3 features in all 4 machine learning models. This could be indicative of the greater predictive value of features that convey the overall tone of a patient portal message, like the sentiment score as opposed to the frequencies of individual words. In this vein, the stemmed keyword “suicid” was not one of the top features converged on by any of the machine learning models. For example, in the random forest model, the keyword “suicid” was the 15th highest weighted feature. (Figure 2) It is possible that this could be because of the keyword “suicid” having a low frequency of mentions in our particular dataset, appearing in only 15 of the 420 messages associated with a 30-day SRE. In larger datasets, which may have greater utilization of the keyword “suicid,” the relevance of this particular keyword as a predictive feature may be higher.

Prediction of suicide risk using patient portal messages like our study confers several advantages over doing so with social media. Patient portal messages are a confidential and secure communication between patients and health care providers; as a result, they may include more sensitive health-related information that patients might be hesitant to share publicly on social media. Moreover, because patient portal messages typically have longer character limits than most social media platforms, patient portal messages may be more likely to include detailed personal narratives, emotional expressions, and specific information about mental health-related symptoms, making them a rich source of data for analysis. Furthermore, patient portal messages provide an ongoing record of patient communications with health care professionals, allowing for longitudinal analysis of changes in language patterns and treatment response. This may allow health care providers and AI to track the progression of SI over time while assessing the response to therapeutic interventions. By leveraging the power of NLP techniques to analyze patient portal messages, future research may uncover previously undetected patterns and digital biomarkers predictive of suicide risk, ultimately paving the way for personalized suicide prevention efforts and enhanced mental health care delivery.

### Limitations

There are several key limitations to keep in mind with interpretation of the present study. First, it is important to note that language models are “data hungry” and often can only achieve optimal performance after being trained on a large volume of cases. The number of messages associated with a 30-day SRE in our study thus becomes a limiting factor in model performance. In that vein, our choice of a “bag-of-words” NLP model also limits model performance compared with state-of-the-art large language models. This choice was deliberate so that we could perform a thorough explainability analysis. Another important limitation is that the present study does not control for clustering, that is, multiple messages from the same patient with their own unique profile of interacting with the patient portal affecting the aggregate analysis. In addition, emotive language expression may be mediated by patient-level demographic characteristics and mood and sociocultural, sex, and geographic factors. As a result, messaging patterns may differ across groups or even within one individual over time. Because our current model was unable to account for all of these factors, future studies should explore the effect of these patient-and context-specific factors on portal messaging patterns. Finally, our dataset was limited by the small number of control messages and completed suicides in our patient cohort. In addition, the results may have been affected by the inclusion of hospital admissions for depression. Future research should validate and build on our results by incorporating other metadata from patient portal messages, such as word count, number of messages, and message timing.

## Conclusion

An NLP AI trained on a subset of patient portal message data from our institution was able to predict 30-day SRE with sensitivity and specificity at a level comparable with commonly used suicide screening stools. Predictors that conveyed the overall tone of a patient message, such as the sentiment score, were more highly weighted by machine learning models in predicting 30-day SRE than the frequencies of individual words. The predictive model developed in this study, which was trained on a subset of patient portal message data can serve as a proof-of-concept for future NLP models trained on larger datasets and specifically designed for suicide prevention.

## Potential Competing Interests

No authors declare a conflict of interest.
